# Inverse association of antioxidant and phytoestrogen nutrient intake with adult glioma in the San Francisco Bay Area: a case-control study

**DOI:** 10.1186/1471-2407-6-148

**Published:** 2006-06-03

**Authors:** Nicole Tedeschi-Blok, Marion Lee, Jennette D Sison, Rei Miike, Margaret Wrensch

**Affiliations:** 1 Department of Preventive Medicine, Keck School of Medicine, University of Southern California, SRT 1015, MS 103, Children's Hospital Los Angeles, Los Angeles, USA; 2 Department of Neurological Surgery, University of California San Francisco, School of Medicine, San Francisco, USA; 3 Department of Epidemiology and Biostatistics, University of California San Francisco, School of Medicine, San Francisco, USA

## Abstract

**Background:**

Increasing evidence from epidemiologic studies suggest that oxidative stress may play a role in adult glioma. In addition to dietary antioxidants, antioxidant and weak estrogenic properties of dietary phytoestrogens may attenuate oxidative stress. Our hypothesis is that long-term consumption of dietary antioxidants and phytoestrogens such as genistein, daidzein, biochanin A, formononetin, matairesinol, secoisolariciresinol and coumestrol, may reduce the risk of adult glioma.

**Methods:**

Using unconditional logistic regression models, we compared quartiles of consumption for several specific antioxidants and phytoestrogens among 802 adult glioma cases and 846 controls from two study series from the San Francisco Bay Area Adult Glioma Study, 1991 – 2000, controlling for vitamin supplement usage, age, socioeconomic status, gender, ethnicity and total daily calories. For cases, dietary information was either self-reported or reported by a proxy. For controls, dietary information was self-reported. Gender- and series- specific quartiles of average daily nutrient intake, estimated from food-frequency questionnaires, were computed from controls.

**Results:**

Significant p-values (trend test) were evaluated using significance levels of either 0.05 or 0.003 (the Bonferroni corrected significance level equivalent to 0.05 adjusting for 16 comparisons). For all cases compared to controls, statistically significant inverse associations were observed for antioxidant index (p < 0.003), carotenoids (alpha- and beta-carotene combined, p < 0.05), daidzein (p = 0.003), matairesinol (p < 0.05), secoisolariciresinol (p < 0.003), and coumestrol (p < 0.003). For self-reported cases compared to controls, statistically significant inverse associations were observed for antioxidant index (p < 0.05) and daidzein (p < 0.05).

**Conclusion:**

Our results support inverse associations of glioma with higher dietary antioxidant index and with higher intake of certain phytoestrogens, especially daidzein.

## Background

Epidemiologic evidence from studies of dietary antioxidant intake and adult glioma support a role for oxidative stress in gliomagenesis. Several studies have shown that antioxidant consumption from fruits and vegetables may be protective against adult glioma [1-4]. Most recently, a large case-control study in Nebraska showed that increased intake of carotenoids reduces the risk of glioma by 50 percent and suggests that antioxidant as well as phytochemical nutrient intake is likely to play a protective role in adult glioma [4]. Pro-inflammatory and pro-angiogenic mediators may also be implicated in glioma development and progression. Recent studies suggest that estrogen receptors may play a role in decreasing both pro-inflammatory and pro-angiogenic mediators [5,6]. Furthermore, estrogen receptor expression is negatively correlated with astrocytic tumor grade [7]. Phytoestrogens, plant-derived chemicals with both antioxidative and estrogenic properties, have structural similarities to 17-beta-estradiol and can be categorized to isoflavones (eg., genistein, daidzein, biochanin A, and formononetin), lignans (eg., matairesinol and secoisolariciresinol), and coumestans (eg., coumestrol). Some foods rich in isoflavones include soy, lentils, and beans. Foods rich in lignans include oilseeds, whole-grain cereals, legumes, and berries; while alfalfa and clover are foods rich in coumestans. In addition to their antioxidative and estrogenic properties, phytoestrogens are noted for their clinical potential in antiviral, antibacterial, antiproliferative, angiogenic activities [8].

If oxidative stress is involved in brain tumor development, then dietary intake of nutrients of fruits and vegetables with antioxidative and estrogenic properties may reduce the risk of adult glioma. We addressed this hypothesis by comparing nutrient intake between incident glioma cases and controls of the San Francisco Bay Area Adult Glioma Study, 1991–2000.

## Methods

Human subject methods were approved by the University of California Committee on Human Research, approval number H6539-04956. Informed consent was documented through subject signature on the approved consent document.

### Case ascertainment

The San Francisco Bay Area Adult Glioma Study, 1991–2000, is described in detail elsewhere [2,9-12]. Briefly, cases were identified within a median of seven weeks of diagnosis using Northern California Cancer Center's rapid case ascertainment system. Eligible cases included those age 20 years and older, with histologically confirmed newly diagnosed glioma, International Classification of Disease for Oncology codes 9380 to 9481, who resided in 6-counties of the San Francisco Bay Area, which included Alameda, Contra Costa, Marin, San Mateo, San Francisco, or Santa Clara. Ascertainment periods included August 1991 to April 1994 (series 1) and from May 1997 to August 2000 (series II). Pathologic specimens were obtained and reviewed by a single neuropathologist in each series [13,14], thereby minimizing misclassification of diagnosis.

### Control ascertainment

In both series, controls were identified by random digit-dialing and frequency matched to cases on ten-year age groups, gender, and ethnicity (White, Black, Hispanic, Asian, or other). Initial sampling units included area code, 3-digit prefix, and 2 digits of cases' phone numbers. Eligible matches were found for each sampling unit through 2 digit suffixes generated from random number tables [2].

### Dietary questionnaires and interviews

#### Series I

Eligible cases or next-of-kin and controls were sent a letter and telephoned to schedule an in-person interview at subjects' home or other location of their choice [2]. Interviews were conducted with consenting subjects or their next-of-kin (proxies) when cases were not available due to death or disability. Subjects were sent a self-administered dietary questionnaire in advance, which was collected or completed at the in-person interview. All questions were asked for the year prior to glioma diagnosis for cases and for the previous year for controls.

The series I questionnaire consisted of 79-item food-frequency questions modified from the US National Cancer Institutes' (Block's) Health Habits and History Questionnaire [15] and the Los Angeles Glioma and Meningioma Study Questionnaire [16]. The modifications were originally made to emphasize antioxidant, nitrite, and nitrate – containing foods [2]. Food items were group into the following categories: fruits and juices; vegetables; breakfast foods; lunch foods; meat, fish, poultry and mixed dishes; breads, salty snacks and spreads; sweets; dairy products and beverages. Participants were asked to choose usual frequencies of consumption of each food item. The frequency choices included: 'never', 'less than 1 per month', '1 per month', '2–3 per month', '1 per week', '2 per week', '3–4 per week', '5–6 per week', '1 per day', and '2 or more per day'. Portion sizes were not asked, but were estimated using gender- and age- specific values from the Block and colleagues' National Cancer Institute's Health Habits and History Questionnaire [15]. Additional questions were asked about how often lemon juice was added to fish, fruit juices or tea was consumed with cured meat or bacon, and beer was consumed with meals. Frequency of vitamin supplement use (never or number of times per day, week, month, or year as well as dose) was also asked.

#### Series II

Similarly, eligible cases or next-of-kin and controls were sent a letter and telephoned to schedule an in-person interview at subjects' home or other location of their choice. Interviews were conducted with consenting subjects or their proxies when cases were not available due to death or disability. Rather than a self-administered diet questionnaire as in series I, interviewers asked the diet questions in series II. As in series I, all questions were asked for the year prior to glioma diagnosis for cases and for the previous year for controls in series II.

The series II diet questionnaire consisted of 96 food-frequency questions modified from the US National Cancer Institutes' (Block's) Health Habits and History Questionnaire [15] and included questions on portion sizes. Modifications included additional fruits, vegetables, and soy products (eg., avocado, banana, fresh peaches, mangoes or papayas, beets, cauliflower, celery, turnips, radishes, rhubarb, salsa, soy burgers, soy milk, and, tofu). The frequency choices included: 'never or less than 1 per month', '1 per month', '2–3 per month, '1 per week', '2 per week', '3–4 per week', '5–6 per week', '1 per day'. Portion sizes were asked according to the number of pieces, or either small, medium, large, or extra-large, for food frequencies of 1 per week or greater; using visual aids and 3-dimensional models. Questions on type (multi-vitamin or antioxidant), frequency and duration of vitamin supplements usage were also included.

#### Series I & II

For the purposes of assessing major differences of dietary consumption comparing eligible participants to eligible non-participants, an abbreviated questionnaire was administered to some unwilling to participate in the full interview. In a 5-minute phone interview, the abbreviated questionnaire asked basic demographic and dietary (including vitamin supplement use) questions.

### Dietary analyses

For both series, only one value was assigned for each food frequency choice by converting the unit of year, month or day to the number of times per day. For ranges of frequencies, the mid-value was assigned before conversion, e.g. 2.5 times per month was assigned if the '2–3 per month' category was given. Intake of each food item was converted to grams consumed per day by applying an appropriate algorithm for each series as described in the following: For series I, the frequency and an assigned gender- and age-specific medium portion size were multiplied against a nutrient database to produce grams consumed per day for each food item. The assigned gender- and age-specific medium portions were based on the Block and colleagues' National Cancer Institute's Health Habits and History Questionnaire Personal Computer System Packet [17]. For series II, the frequency and portion reported were multiplied against the same nutrient database to produce grams consumed per day for each food item. For both series, this nutrient database, which included a total of 27 nutrients per 100 grams for each food item, was based on the California Teacher's Study [18,19], USDA nutrient database [20], and the Block and colleagues' National Cancer Institute's Health Habits and History Questionnaire Personal Computer System Packet [17]. The summation of nutrient amounts over all food items provided a subject's total daily intake for 27 nutrients. Of the 27 nutrients, there was a total of 15 antioxidants and phytoestrogens for our case-control comparisons.

Additionally for both series, a total daily antioxidant index intake was calculated by summing the product of grams consumed over all food items and units of antioxidant index per gram from an antioxidant index database for fruit, vegetable, juice and tea food items. We constructed the antioxidant index database based on data from Wang and colleagues [21] (for fruits and fruit juices), Cao and colleagues [22] (for fruits and vegetables) and Rice-Evans and Miller [23] (for fruits, vegetables, and teas). A total of 35 items of fruits, vegetables, juices and teas from either series were included in the antioxidant index database. All 35 food items were available for series II participants, while only 22 of the 35 food items were available for series I participants (see above for additional fruits and vegetables included in series II only). Therefore, because the antioxidant index was the sum of antioxidant values across food items reportedly consumed by each subject, series II participants had higher values for the total antioxidant index than series I participants. Antioxidant index values were units of micromoles Trolox equivalents per gram of food (fruits, vegetables, juices or teas). Trolox equivalents per gram of food were measured by the oxygen radical absorbance capacity (ORAC) assay [21,22]. The ORAC assay measures the degree to which a sample inhibits oxidizing agent action using Trolox, a water-soluble analogue of vitamin E, as a control standard.

SAS version 8.02 (SAS Institute Inc, Cary, NC) was used for all statistical analyses. Demographic distributions of cases were compared to those of controls using t-tests or analysis of variance (ANOVA) for continuous variables and chi-square tests for categorical variables. Means and standard errors as well as geometric means with upper and lower 95% confidence limits for daily consumption of total antioxidant index as well as various individual antioxidants and phytoestrogens adjusted for total calories were generated with general linear models for all cases, self-reporting cases and controls using SAS procedure GLM. Logistic regression models estimated odds ratios for quartiles of consumption of antioxidant index or each nutrient adjusting for age (continuous), gender, ethnicity (White versus other), socioeconomic status (combined income and education categories), and total calories (continuous). Thus, to adjust for total energy intake, we employed the standard multivariate method [24]. In addition, vitamin supplement use and/or meat consumption were explored as covariates in the logistic regression models.

Case-control comparisons were analyzed separately by series since data on nutrient and antioxidant index intake was collected by different methods between study series. Because comparison of series-specific odds ratios and corresponding 95% confidence showed that odds ratios were largely in the same direction and their corresponding 95% confidence intervals overlapped between series, we also conducted combined series case-control comparisons with a study series indicator variable as a necessary covariate in logistic regression models. Gender- and series- specific quartile cutpoints were determined for the antioxidant index or nutrient intake levels based on food consumption among the controls. Test for trend were computed for case-control odds ratios of antioxidant index or nutrient quartiles. Results are described for p-values generated based on a 5% significance level and two-sided hypothesis tests. Finally, a Bonferroni correction for 16 simultaneous comparisons (an antioxidant index plus 15 nutrients) was applied to emphasize results based on a 0.003 (= 0.05/16) significance level.

## Results

The total number of subjects from both series that were identified as eligible to participate in the San Francisco Bay Area Adult Glioma Study, 1991–2000, includes 1,110 cases and 1,284 controls. Participation was greater among eligible cases (79%–80% for series I and II) than among eligible controls (67% for combined series data, 74% for series I, and 60% for series II) [9-11]. Dietary data for 98 percent (846/864) of controls, 94 (497/524) percent of self-reported cases and 87 (305/349) percent of proxy-reported cases were included in the dietary analyses, based on the number of items with missing food frequency and daily intake of total calories. Table 1 summarizes frequency distributions for vitamin supplement intake, demographics, and clinical factors for 802 cases (including responses from 305 proxies and 497 self-reporting cases) and 846 controls included in the dietary analyses. Because of better survival of younger cases, the mean age for self-reported cases was 50 years, significantly lower than that of the controls (p < 0.001, table 1). For combined income and education, self-reported cases had greater socioeconomic status than controls (p < 0.001, table 1); likely due to younger ages of self-reporting cases. Geometric means of total daily nutrients consumed adjusted for total calories among cases and controls are shown in table 2. The observed average daily nutrients consumed were reasonable values for the U.S.

Information on vitamin type (multi-vitamin or antioxidant), asked for series II, was not considered during the analyses because the reported data did not appear to reflect a reasonable distribution of supplement types. In addition, differential absorption rates between supplements and foods prevent the combination of nutrients from supplement types with that from foods consumed. Therefore, all vitamin supplement analyses only considered intake (yes or no). Results of an abbreviated diet questionnaire for comparisons of participants and non-participants, showed that vitamin use among controls adjusted for age, gender, and ethnicity was similar for participants and non-participants (OR = 1.3, 95% CI = 0.91 to 1.82; data not shown). Interestingly, the percent of cases (among all and self-reported cases) that regularly consumed vitamin supplements was significantly lower than that of controls (p < 0.001, table 1). Regardless of study series, a significantly lower percentage of cases (either all or self-reported) than controls reported use of vitamin supplements (p = 0.002 and p = 0.005 for series I and II, respectively; data not shown). For this reason, we also compared case-control nutrient consumption stratified by vitamin supplement use and observed a high degree of overlap among 95% confidence intervals of odds ratios across strata (data not shown).

Age- gender-, ethnicity-, socioeconomic status-, and supplement intake-adjusted odds ratios for quartiles of total calories consumed are shown in table 3. Case-control odds ratios for quartiles of nutrient intakes adjusted for age, gender, ethnicity, socioeconomic status, total calories, and supplement intake are shown in tables 4 and 5. Case-control distributions (= N) for quartiles of nutrient consumption are provided in supplemental table 1 [see Additional file 1]. Examining these results for those that were statistically significant and consistent for all cases versus controls and self-reported cases versus controls, we found that odds ratios for intakes of antioxidant index, carotenoids (alpha- and beta-carotene combined), and the phytoestrogens, daidzein, and matairesinol were reduced (tables 4 and 5). Although not consistent with self-reported cases compared to controls, other notable findings regarding nutrient intake include statistically significant differences among all cases compared to controls, which include consumption of vitamin C, and the phytoestrogens, genistein, formononetin, secoisolariciresinol and coumestrol (table 4). After applying a Bonferroni correction, statistically significant p-values for trend tests were observed for all cases compared to controls among combined series data for antioxidant index (p = 0.002), daidzein (p = 0.003), secoisolariciresinol (p = 0.001) and coumestrol (p = 0.001). Additionally, after adjustment for meat consumption, the only noteworthy changes were for vitamin C consumption. Consumption of vitamin C for cases was no longer significantly different than that of controls after adjusting for meat consumption (OR = 0.77, 95% CI = 0.58 to 1.02, for second quartile compared to lowest; OR = 0.78, 95% CI = 0.59 to 1.05, for third quartile compared to lowest; OR = 0.76, 95% CI = 0.56 to 1.04, for highest quartile compared to lowest; combined series I and II for all cases compared to controls). The effect of adjustment for meat consumption was negligible for all other dietary variables.

## Discussion

We previously discussed in detail major potential sources of bias in these case-control dietary data [2]. Briefly, bias could result from differential data quality between proxy and self-reported dietary histories with more accurate data from self-reports. Differential participation of controls who are more health conscious and therefore might eat foods that are considered to be healthier such as fruits and vegetables could also bias results. In addition, the dietary data collected may not accurately represent actual dietary data during the etiologically relevant period of brain tumor development; however, this etiologically relevant time period is unknown. For this reason, the period of dietary recall was limited to recent dietary habits to improve recall accuracy. Furthermore, non-differential dietary misclassification may affect series I data as a result of using computed measures of dietary nutrients because these computations assume consumption of average portion sizes for age group and gender specific subjects, which may decrease detectable differences in nutrient consumption. This issue is likely to be overcome in series II data, which ascertained portion sizes for foods consumed once a week or more. Also, since actual nutrient values of foods depend on soil quality and other factors, the nutrient database values used in these analyses may either over or underestimate the actual nutrients of the foods consumed. Since, to our knowledge, this is the first study to investigate an association between antioxidant index of foods consumed with a disease, we used estimates for antioxidant values based solely on literature review. Our findings of an inverse association between antioxidant index and glioma may encourage other researchers to further investigate these antioxidant indices and their physiologic relevance. In addition, since the series I food-frequency questionnaire lacked some foods with high quantities of phytoestrogens (such as tofu and soy, which are both high in genistein and daidzein), the estimated daily intake of such phytoestrogens are likely lower than actual values. While the series II food-frequency questionnaire included these additional food items that are likely to more accurately assess total intakes of phytoestrogens, evidence of an association is strong in both series data for nutrients driven largely by soy products (especially daidzein). Therefore, we are encouraged that we likely have an overall improved assessment of phytoestrogen intake with consideration of series II data. It should also be noted that individual variability is likely to contribute to differences in metabolism and bioavailability of dietary phytoestrogens [25]. Regarding the validity of the estimates of nutrients consumed for the food-frequency information assessed in this study, both antioxidant [26] and phytoestrogen (Horn-Ross, P. et al., *Am J Epidemiol*, in review, 2005) nutrients estimates closely approximate reference values in previous studies. Finally, although we report findings that were statistically significant at the 0.05 level, because of multiple comparisons, the actual p-values are likely to be higher than those calculated. For this reason, we also emphasize statistically significant results at the 0.003 level after applying a Bonferroni correction for 16 simultaneous comparisons.

Due to methodological differences in data collection between series, we presented the data separately by series. Because the direction and 95% confidence limits of odds ratios were similar across series, we also performed combined series analyses since increasing the sample size increases the power to detect statistically significant findings. It should be noted that our combined series analysis is similar to a meta-analysis, a common epidemiologic practice, with the advantage of having the raw data to directly estimate associations from the combined data. Combined series odds ratios were generated with addition of a series indicator covariate in the logistic regression models. Additionally, because of the many limitations with retrospective dietary assessment, we focus on results that were reasonably consistent in magnitude and statistical significance between all cases versus controls and self-reporting cases versus controls. For those nutrients with such consistent findings, the odds ratios for highest to lowest quartiles of intake tended to be further from the null among subjects without reported supplement use than those with such use. These findings could indicate that distributions of dietary choices of people taking supplements are more similar regardless of case-status.

Despite the many caveats inherent in retrospective dietary assessment, our findings, in light of previous epidemiologic and mechanistic studies, support the growing evidence of a role for oxidative stress in adult glioma. Vitamin C is a water soluble, well known scavenger of hydroxyl radicals that inhibits oxidative DNA lesions such as 8-hydroxydeoxyguanosine [27]. We observed an inverse association with adult glioma for vitamin C consumption, which is consistent with our previous results [2]. Regarding consumption of vitamin E, a lipid soluble free radical scavenger, we did not observe any statistically significantly differences between cases and controls, although total dietary antioxidant index was statistically significantly inversely associated with glioma in this study. However, since results from other studies regarding the roles of vitamin's C and E with brain tumor risk are inconsistent [1,3,28-30], the roles of antioxidant vitamins C & E may remain unclear. If the findings of inverse associations of total antioxidant intake from all fruits, vegetables, juices and teas with glioma are replicated it may suggest a protective role of these compounds for adult glioma.

For carotenoid consumption, more consistent results have been observed among studies of brain tumor risk. We observed an inverse association of carotenoid consumption with glioma risk, which is consistent with other studies [3,28] including a recent study in Nebraska, which showed a two-fold reduction in risk of glioma with increased carotenoid intake [4]. Carotenoids are lipophilic molecules with antioxidant properties implicated in scavenging peroxynitrite, modulating DNA repair [31], and possibly anti-inflammatory mechanisms [32-35].

We observed statistically significant inverse associations for consumption of several phytoestrogens suggesting protective effects. The most consistently significant inverse association was observed for daidzein. Our results also suggest protective effects against gliomas for formononetin, matairesinol, secoisolariciresinol, and coumestrol. Vaya and colleagues [36] showed that flavonoids, which include isoflavones and coumestans, inhibit low density lipoprotein oxidation. More specifically, mechanistic studies suggest that flavonoids may down-regulate both the expression of iNOS and cyclooxygenase-2 (COX-2), both of which have pro-inflammatory roles [37-39]. Epidemiologic findings also support a role for inflammatory and immune mediators in brain tumors. An inverse association of brain tumors with asthma and autoimmune disease has been observed [10,40], which might partly be related to an inverse association of non-steroidal anti-inflammatory drug (NSAID) use or other anti-inflammatory drugs [41].

Furthermore, the influence of phytoestrogens on pro-inflammatory mediators may be a result of estrogen receptor-dependent activities. Recent evidence from several studies supports that estrogen receptors, and especially estrogen receptor-beta, down-regulates iNOS and COX-2 gene expression [5,6]. Interestingly, expression of estrogen receptor-beta has been characterized in astrocytic tumors, showing that as tumor grade increases, estrogen receptor-beta expression decreases [7]. Furthermore, Hara and Okayasu recently showed a strong correlation of vascular endothelial growth factor (VEGF) and COX-2 expression, likely due to increased iNOS expression, with degree of angiogenesis in astrocytomas [42]. Could phytoestrogen intake play a role in preventing the progression of astrocytic tumors from low to high grade through estrogen receptor-dependent down-regulation of pro-inflammatory and pro-angiogenic mediators? Further studies of phytoestrogen intake and astrocytic tumors are necessary to clarify the suggestive association.

## Conclusion

The observed inverse associations suggest a protective role against gliomagenesis for consumption of foods rich in antioxidants and certain phytoestrogens, especially daidzein. In light of the growing evidence supporting a role for oxidative stress in gliomagenesis, future studies in brain tumor research should focus on reactive nitrogen and oxygen species to further clarify their roles as well as identifying targets of treatment and prevention of glioma.

## Competing interests

The author(s) declare that they have no competing interests.

## Authors' contributions

We, the authors, are noted for the following significant contributions toward this study: ML for the overall idea; MW and ML for the study design; MW and RM for the collection of data; JDS and RM for analysis of data; NTB for writing the manuscript; and both ML and MW for advice and consultation.

## Pre-publication history

The pre-publication history for this paper can be accessed here:



## Supplementary Material

Additional File 1 Supplmental_Table1, "Case-control distributions for quartiles of nutrient consumption; San Francisco Bay Area Adult Glioma Study, 1991–2000", 133 KB Click here for file

## Figures and Tables

**Table 1:** Distribution of vitamin supplement intake, age, gender, ethnicity, income, education, body mass index and tumor type for eligible participants for combined series I & II; San Francisco Bay Area Adult Glioma Study, 1991–2000

Description		Cases^1^					
		
	Controls (N = 846)	All (N = 802)		Self-reported (N = 497)		Proxy-reported (N = 305)	
Vitamin Supplement intake (% YES)	62.6	52.4	***	50.2	***	56.0	*
*General Demographics*							
Age (median) ± SE^2^	55.1 ± 0.6	55.4 ± 0.6	ns	49.7 ± 0.7	***	64.7 ± 0.8	***
Gender as % Male	54.5	57.1	ns	58.6	ns	54.8	ns
Ethnicity as % White^3^	83.8	83.3	ns	83.9	ns	82.3	ns
*Socio-economic Status Measures (%)*							
Household Income^4^							
<$29,999	25.0	24.3	ns	17.4	***	36.0	***
$30,000–$69,999	40.8	39.8		38.5		42.1	
≥$70,000	34.1	35.9		44.1		21.9	
Highest Education							
Less than high school	6.0	10.1	**	4.8	ns	18.7	***
High school, some college	48.5	48.9		47.5		51.2	
College graduate and/or more	45.5	41.0		47.7		30.2	
Socio-economic Status^5^							
<High School education or income <$29,999	27.3	27.7	ns	19.1	***	41.6	***
≥High School graduate or income $30,000–$69,999	53.2	50.4		52.7		46.6	
College graduate and income ≥ $70,000	19.5	22.0		28.2		11.8	
Body Mass Index^6 ^(median) ± SE^2^	25.7 ± 0.2	25.9 ± 0.2	ns	25.7 ± 0.2	ns	26.2 ± 0.3	ns
Tumors Types (%)							
Glioblastoma		59.2		47.7		78.0	
Anaplastic Astrocytoma		11.2		10.7		12.1	
Astrocytoma		5.4		7.2		2.3	
Oligodendroglioma		11.1		16.5		2.3	
Oligoastrocytoma		6.2		8.5		2.6	
Ependymoma		1.0		1.4		0.3	
Other		5.9		8.1		2.3	

^1 ^p-values for case-control comparisons (t-tests for continuous variables or chi-square tests for categorical variables): * p = 0.05, **p = 0.01, ***p = 0.001, or not significant (ns). ^2 ^SE = standard error. ^3 ^The category other than white includes African-Americans, Chinese, Japanese, other Asians, Filipinos, Mexicans, other Latinos, and others. ^4 ^Percentages do not total 100% due to "Refuse to Answer" and "Don't Know". ^5 ^Categories for Socio-economic Status combine highest education attained and/or household income range. ^6 ^BMI calculated from reported height and weight at interview.

**Table 2:** Geometric means of consumption of daily nutrients adjusted for total calories*; San Francisco Bay Area Adult Glioma Study, 1991–2000.

**NUTRIENT**	**Controls (N = 846)**	**All Cases (N = 802)**	**Self-reported Cases (N = 497)**
Antioxidant index^1^	2675.3	2433.3	2450.4
Vitamin E (mg)	7.8	7.7	7.6
Vitamin C (mg)	115.8	107.6	109.6
Carotenoids^2 ^(mcg)	3743.3	3404.0	3327.1
Alpha-carotene (mcg)	672.6	581.4	554.6
Beta-carotene (mcg)	3008.8	2756.0	2697.7
Lutein (mcg)	2094.3	1884.7	1911.4
Lycopene (mcg)	1324.4	1310.6	1320.9
Cryptoxanthin (mcg)	114.1	106.7	111.0
*Phytoestrogens:*			
Formononetin (mcg)	14.7	14.2	15.2
Coumestrol (mcg)	121.1	107.9	112.1
Matairesinol (mcg)	25.3	23.3	23.6
Genistein (mcg)	260.4	233.5	253.9
Daidzein (mcg)	396.9	352.7	372.8
Biochanin A (mcg)	25.4	23.5	24.0
Secoisolariciresinol (mcg)	115.0	104.1	103.5
Total Protein (g)	60.9	59.7	59.2
Total Fat (g)	56.4	58.2	56.8
Total Cholesterol (mg)	200.7	210.2	204.7
Total Kcal (unadjusted)	1609.6	1646.5	1612.7

^1^Units are daily micromoles of Trolox equivalents gram of fresh weight as determined by the oxygen radical absorbance capacity (ORAC) assay. ^2 ^alpha- and beta-Carotene. *Note: Means within each group were adjusted for total calories.

**Table 3:** Odds ratios for cases versus controls by quartiles of total calories adjusted for age, gender, ethnicity, SES*, and supplement use; San Francisco Bay Area Adult Glioma Study, 1991–2000.^†^

**Quartiles of Total Calories^2^**	**Combined Series I & II^1^**				**Series I (1991–1995)**				**Series II (1996–2000)**			
	
	**OR**	**95% CI^3^**		**p^4^**	**OR**	**95% CI^3^**		**p^4^**	**OR**	**95% CI^3^**		**p^4^**
**All case-control**												
1st	1	--	--		1	--	--		1	--	--	
2nd	1.27	0.87	1.84	0.51	1.49	0.87	2.54	0.45	1.07	0.63	1.82	0.93
3rd	1.15	0.70	1.90		1.41	0.69	2.88		0.93	0.46	1.90	
4th	1.47	0.72	3.02		1.79	0.65	4.97		1.21	0.43	3.39	
**Self-reported case-control**												
1st	1	--	--		1	--	--		1	--	--	
2nd	1.22	0.79	1.87	0.74	1.62	0.84	3.11	0.53	0.98	0.55	1.76	0.83
3rd	1.11	0.62	1.99		1.59	0.66	3.83		0.82	0.37	1.80	
4th	1.30	0.56	2.99		1.79	0.51	6.23		1.03	0.32	3.26	

*Categories for socio-economic status (SES) combine highest education attained and/or household income range. ^†^Case-control distributions (= N) for quartiles of nutrient consumption are provided in supplemental table 1 [see Additional file 1]. ^1^Also adjusted for study series.^2^Quartile cutpoints were calculated from series- and gender-specific nutrient intake distributions among controls. ^3^CI = Confidence interval. ^4 ^p-value for chi-square test for trend.

**Table 4:** Odds ratios for all glioma cases versus controls by quartiles of daily nutrient intake adjusted for age, gender, ethnicity, SES*, total calories**, and supplement use; San Francisco Bay Area Adult Glioma Study, 1991–2000.^†^

**Nutrient Quartile^2^**	**Combined Series I & II^1^**				**Series I (1991–1995)**				**Series II (1996–2000)**			
	
	**OR**	**95% CI^3^**		**p^4^**	**OR**	**95% CI^3^**		**p^4^**	**OR**	**95% CI^3^**		**p^4^**
**Antioxidant Index**												
1st	1	--	--	**0.002**	1	--	--	**0.004**	1	--	--	0.12
2nd	0.81	0.61	1.07		0.82	0.56	1.20		0.79	0.53	1.20	
3rd	0.82	0.62	1.09		0.77	0.52	1.14		0.89	0.59	1.35	
4th	**0.57**	**0.42**	**0.78**		**0.52**	**0.33**	**0.79**		0.64	0.40	1.03	
**Vitamin E**												
1st	1	--	--	0.45	1	--	--	0.46	1	--	--	0.06
2nd	0.95	0.71	1.27		1.12	0.74	1.69		0.80	0.53	1.22	
3rd	0.79	0.57	1.10		1.02	0.64	1.62		**0.60**	**0.37**	**0.96**	
4th	0.91	0.62	1.34		1.28	0.75	2.19		0.62	0.35	1.09	
**Vitamin C**												
1st	1	--	--	**0.02**	1	--	--	**0.03**	1	--	--	0.31
2nd	**0.74**	**0.56**	**0.97**		0.75	0.51	1.09		0.72	0.48	1.09	
3rd	**0.73**	**0.55**	**0.97**		0.71	0.48	1.05		0.75	0.49	1.14	
4th	**0.70**	**0.51**	**0.94**		**0.63**	**0.41**	**0.95**		0.78	0.50	1.23	
**Carotenoids^5^**												
1st	1	--	--	**0.02**	1	--	--	0.23	1	--	--	**0.03**
2nd	**0.72**	**0.54**	**0.95**		0.73	0.49	1.08		0.71	0.48	1.06	
3rd	0.84	0.63	1.11		0.93	0.64	1.37		0.74	0.49	1.11	
4th	**0.65**	**0.48**	**0.88**		0.70	0.46	1.06		**0.60**	**0.39**	**0.93**	
**α-Carotene**												
1st	1	--	--	0.07	1	--	--	0.46	1	--	--	0.07
2nd	0.87	0.66	1.15		1.03	0.70	1.52		0.73	0.49	1.09	
3rd	0.83	0.63	1.10		1.06	0.72	1.58		**0.64**	**0.42**	**0.96**	
4th	0.76	0.57	1.02		0.83	0.55	1.27		0.71	0.47	1.08	
**β-Carotene**												
1st	1	--	--	**0.04**	1	--	--	0.24	1	--	--	0.09
2nd	0.79	0.60	1.04		0.73	0.49	1.08		0.85	0.58	1.27	
3rd	0.79	0.59	1.05		0.86	0.58	1.27		0.70	0.46	1.06	
4th	**0.72**	**0.54**	**0.98**		0.73	0.48	1.10		0.72	0.47	1.11	
**Lutein**												
1st	1	--	--	0.06	1	--	--	**0.05**	1	--	--	0.55
2nd	1.02	0.78	1.34		1.17	0.81	1.70		0.86	0.57	1.29	
3rd	0.86	0.65	1.14		0.88	0.60	1.30		0.85	0.56	1.28	
4th	0.79	0.59	1.05		0.71	0.47	1.07		0.88	0.58	1.33	
**Lycopene**												
1st	1	--	--	0.75	1	--	--	0.98	1	--	--	0.69
2nd	0.91	0.69	1.21		0.99	0.67	1.46		0.83	0.55	1.25	
3rd	1.01	0.76	1.33		1.05	0.72	1.55		0.96	0.63	1.45	
4th	1.02	0.76	1.36		0.98	0.66	1.48		1.05	0.68	1.60	
**Cryptoxanthin**												
1st	1	--	--	0.08	1	--	--	0.12	1	--	--	0.39
2nd	0.96	0.73	1.26		0.76	0.52	1.11		1.26	0.84	1.88	
3rd	0.92	0.69	1.21		0.86	0.59	1.26		1.00	0.65	1.52	
4th	0.77	0.57	1.03		0.69	0.46	1.02		0.88	0.56	1.38	
**Genistein**												
1st	1	--	--	0.06	1	--	--	0.20	1	--	--	0.11
2nd	0.77	0.58	1.02		**0.65**	**0.43**	**0.97**		0.90	0.60	1.35	
3rd	**0.71**	**0.52**	**0.96**		**0.56**	**0.36**	**0.87**		0.89	0.58	1.36	
4th	0.74	0.54	1.02		0.74	0.45	1.20		0.68	0.44	1.06	
**Daidzein**												
1st	1	--	--	**0.003**	1	--	--	0.30	1	--	--	**0.0006**
2nd	0.77	0.58	1.01		0.96	0.65	1.41		**0.60**	**0.40**	**0.90**	
3rd	**0.61**	**0.46**	**0.82**		**0.65**	**0.43**	**0.98**		**0.56**	**0.37**	**0.86**	
4th	**0.67**	**0.50**	**0.90**		0.90	0.59	1.38		**0.47**	**0.30**	**0.72**	
**Biochanin A**												
1st	1	--	--	0.10	1	--	--	**0.04**	1	--	--	0.84
2nd	0.84	0.63	1.11		**0.64**	**0.43**	**0.95**		1.12	0.75	1.68	
3rd	0.77	0.58	1.02		0.71	0.48	1.05		0.82	0.54	1.26	
4th	0.79	0.59	1.07		**0.61**	**0.40**	**0.92**		1.06	0.69	1.65	
**Formononetin**												
1st	1	--	--	0.58	1	--	--	0.58	1	--	--	0.81
2nd	0.76	0.57	1.00		0.73	0.50	1.08		0.78	0.52	1.18	
3rd	**0.69**	**0.52**	**0.92**		**0.63**	**0.42**	**0.93**		0.77	0.51	1.17	
4th	0.95	0.72	1.27		0.94	0.64	1.39		0.96	0.63	1.47	
**Matairesinol**												
1st	1	--	--	**0.03**	1	--	--	**0.01**	1	--	--	0.60
2nd	**0.74**	**0.56**	**0.98**		**0.59**	**0.40**	**0.86**		0.95	0.64	1.42	
3rd	0.80	0.60	1.06		0.78	0.54	1.15		0.80	0.53	1.22	
4th	**0.68**	**0.50**	**0.92**		**0.50**	**0.33**	**0.77**		0.93	0.60	1.46	
**Secoisolarici-resinol**												
1st	1	--	--	**0.001**	1	--	--	**0.03**	1	--	--	**0.003**
2nd	1.01	0.77	1.33		0.74	0.50	1.08		1.37	0.93	2.03	
3rd	0.81	0.61	1.08		0.80	0.55	1.17		0.80	0.52	1.23	
4th	**0.60**	**0.44**	**0.82**		**0.60**	**0.40**	**0.92**		**0.57**	**0.35**	**0.91**	
**Coumestrol**												
1st	1	--	--	**0.001**	1	--	--	**0.01**	1	--	--	**0.02**
2nd	**0.71**	**0.54**	**0.94**		0.73	0.49	1.07		0.68	0.45	1.01	
3rd	**0.62**	**0.46**	**0.83**		**0.60**	**0.40**	**0.90**		**0.62**	**0.41**	**0.94**	
4th	**0.59**	**0.43**	**0.80**		**0.57**	**0.37**	**0.88**		**0.59**	**0.37**	**0.93**	

*Categories for socio-economic status (SES) combine highest education attained and/or household income range. **Total calories = ln(total calories). ^†^Case-control distributions (= N) for quartiles of nutrient consumption are provided in supplemental table 1 [see Additional file 1]. ^1^Also adjusted for study series. ^2^Quartile cutpoints were calculated from series- and gender-specific nutrient intake distributions among controls. ^3^CI = Confidence interval. ^4 ^p-value for chi-square test for trend. ^5 ^alpha- and beta-Carotene. **Note: **Boxed values are statistically significant odds ratios (with corresponding 95% confidence intervals that exclude 1.00) or tests for trend.

**Table 5:** Summary of significant findings for odds ratios among self-reported glioma cases versus controls by quartiles of daily nutrient intake adjusted for age, gender, ethnicity, SES*, total calories**, and supplement use; San Francisco Bay Area Adult Glioma Study, 1991–2000.^†^

**Nutrient Quartile^2^**	**Combined Series I & II^1^**				**Series I (1991–1995)**				**Series II (1996–2000)**			
	
	**OR**	**95% CI**^**3**^		**p^4^**	**OR**	**95% CI^3^**		**p^4^**	**OR**	**95% CI^3^**		**p^4^**
**Antioxidant Index**												
1st	1	--	--	0.06	1	--	--	**0.03**	1	--	--	0.51
2nd	1.08	0.78	1.48		1.06	0.68	1.67		1.05	0.66	1.67	
3rd	1.01	0.73	1.40		0.90	0.56	1.44		1.11	0.70	1.76	
4th	**0.68**	**0.47**	**0.99**		**0.56**	**0.33**	**0.96**		0.79	0.47	1.33	
**Carotenoids^5^**												
1st	1	--	--	0.23	1	--	--	0.31	1	--	--	0.44
2nd	**0.71**	**0.51**	**0.97**		**0.62**	**0.39**	**0.98**		0.79	0.51	1.24	
3rd	0.91	0.66	1.25		0.81	0.51	1.29		0.98	0.63	1.54	
4th	0.74	0.52	1.05		0.71	0.43	1.16		0.76	0.46	1.24	
**β-Carotene**												
1st	1	--	--	0.54	1	--	--	0.45	1	--	--	0.85
2nd	0.77	0.56	1.07		**0.61**	**0.38**	**0.98**		0.96	0.61	1.50	
3rd	0.87	0.63	1.21		0.78	0.49	1.24		0.96	0.60	1.53	
4th	0.87	0.61	1.23		0.78	0.47	1.28		0.95	0.59	1.55	
**Daidzein**												
1st	1	--	--	0.06	1	--	--	0.99	1	--	--	**0.01**
2nd	0.73	0.53	1.01		0.86	0.54	1.38		0.66	0.42	1.04	
3rd	**0.67**	**0.48**	**0.93**		0.76	0.47	1.23		**0.62**	**0.38**	**0.99**	
4th	0.74	0.53	1.04		1.06	0.64	1.77		**0.55**	**0.34**	**0.89**	
**Matairesinol**												
1st	1	--	--	0.32	1	--	--	0.11	1	--	--	0.82
2nd	0.82	0.59	1.13		0.65	0.41	1.03		1.04	0.66	1.63	
3rd	1.02	0.74	1.41		1.00	0.64	1.57		1.04	0.65	1.66	
4th	0.76	0.53	1.09		**0.53**	**0.31**	**0.90**		1.06	0.64	1.75	

*Categories for socio-economic status (SES) combine highest education attained and/or household income range. **Total calories = ln(total calories). ^†^Case-control distributions (= N) for quartiles of nutrient consumption are provided in supplemental table 1 [see Additional file 1]. ^1^Also adjusted for study series. ^2^Quartile cutpoints were calculated from series- and gender-specific nutrient intake distributions among controls. ^3^CI = Confidence interval. ^4 ^p-value for chi-square test for trend. ^5 ^alpha- and beta-Carotene. **Note: **Boxed values are statistically significant odds ratios (with corresponding 95% confidence intervals that exclude 1.00) or tests for trend.
