# Supplementary material for: Inverse association of antioxidant and phytoestrogen nutrient intake with adult glioma in the San Francisco Bay Area: a case-control study
Source: BMC Cancer. 2006 Jun 3;6:148. doi: 10.1186/1471-2407-6-148 (PMC1513391; doi:10.1186/1471-2407-6-148)
Supplement: Additional File 1 — Supplmental_Table1, "Case-control distributions for quartiles of nutrient consumption; San Francisco Bay Area Adult Glioma Study, 1991–2000", 133 KB [file 1471-2407-6-148-S1.doc]

# Additional files

**Supplemental Table 1:** Case-control distributions for quartiles of nutrient consumption; San Francisco Bay Area Adult Glioma Study, 1991-2000.

| **Nutrient Quartile^1^** | **Combined Series I & II** | | | **Series I (1991-1995)** | | | **Series II (1996-2000)** | | |
| --- | --- | --- | --- | --- | --- | --- | --- | --- | --- |
|  | **Controls** | **Cases** | | **Controls** | **Cases** | | **Controls** | **Cases** | |
|  |  | **All** | **Self^2^** |  | **All** | **Self^2^** |  | **All** | **Self^2^** |
| **Antioxidant Index**  1st | 212 | 234 | 145 | 113 | 123 | 71 | 99 | 111 | 74 |
| 2nd | 211 | 193 | 131 | 113 | 110 | 70 | 98 | 83 | 61 |
| 3rd | 211 | 210 | 130 | 113 | 109 | 59 | 98 | 101 | 71 |
| 4th | 211 | 153 | 86 | 113 | 75 | 36 | 98 | 78 | 50 |
| **Vitamin E**  1st | 213 | 203 | 128 | 114 | 96 | 58 | 99 | 107 | 70 |
| 2nd | 211 | 207 | 150 | 113 | 109 | 78 | 98 | 98 | 72 |
| 3rd | 210 | 171 | 100 | 112 | 94 | 46 | 98 | 77 | 54 |
| 4th | 211 | 209 | 114 | 113 | 118 | 54 | 98 | 91 | 60 |
| **Vitamin C**  1st | 212 | 242 | 152 | 113 | 128 | 79 | 99 | 114 | 73 |
| 2nd | 212 | 181 | 118 | 114 | 101 | 59 | 98 | 80 | 59 |
| 3rd | 210 | 182 | 112 | 112 | 96 | 53 | 98 | 86 | 59 |
| 4th | 211 | 185 | 110 | 113 | 92 | 45 | 98 | 93 | 65 |
| **Carotenoids^3^**  1st | 212 | 238 | 163 | 113 | 117 | 82 | 99 | 121 | 81 |
| 2nd | 211 | 178 | 108 | 113 | 91 | 49 | 98 | 87 | 59 |
| 3rd | 211 | 206 | 124 | 113 | 118 | 58 | 98 | 88 | 66 |
| 4th | 211 | 168 | 97 | 113 | 91 | 47 | 98 | 77 | 50 |
| **α-Carotene**  1st | 212 | 221 | 157 | 113 | 101 | 73 | 99 | 120 | 84 |
| 2nd | 211 | 198 | 119 | 113 | 110 | 59 | 98 | 88 | 60 |
| 3rd | 211 | 192 | 106 | 113 | 114 | 57 | 98 | 78 | 49 |
| 4th | 211 | 179 | 110 | 113 | 92 | 47 | 98 | 87 | 63 |
| **β-Carotene**  1st | 212 | 230 | 156 | 113 | 118 | 82 | 99 | 112 | 74 |
| 2nd | 211 | 190 | 115 | 113 | 93 | 49 | 98 | 97 | 66 |
| 3rd | 211 | 189 | 114 | 113 | 110 | 55 | 98 | 79 | 59 |
| 4th | 211 | 181 | 107 | 113 | 96 | 50 | 98 | 85 | 57 |
| **Lutein**  1st | 212 | 212 | 132 | 113 | 108 | 60 | 99 | 104 | 72 |
| 2nd | 211 | 217 | 143 | 113 | 129 | 77 | 98 | 88 | 66 |
| 3rd | 211 | 188 | 114 | 113 | 100 | 58 | 98 | 88 | 56 |
| 4th | 211 | 173 | 103 | 113 | 80 | 41 | 98 | 93 | 62 |
| **Lycopene**  1st | 212 | 197 | 133 | 113 | 100 | 65 | 99 | 97 | 68 |
| 2nd | 211 | 183 | 117 | 113 | 101 | 59 | 98 | 82 | 56 |
| 3rd | 211 | 203 | 113 | 113 | 110 | 56 | 98 | 93 | 57 |
| 4th | 211 | 207 | 129 | 113 | 106 | 56 | 98 | 101 | 73 |
| **Cryptoxanthin**  1st | 212 | 216 | 139 | 113 | 124 | 75 | 99 | 92 | 64 |
| 2nd | 211 | 205 | 123 | 113 | 94 | 53 | 98 | 111 | 70 |
| 3rd | 211 | 198 | 124 | 113 | 109 | 58 | 98 | 89 | 66 |
| 4th | 211 | 171 | 106 | 113 | 90 | 50 | 98 | 81 | 56 |

Supplemental Table 1 (continued).

| **Nutrient Quartile^1^** | **Combined Series I & II** | | | **Series I (1991-1995)** | | | **Series II (1996-2000)** | | |
| --- | --- | --- | --- | --- | --- | --- | --- | --- | --- |
|  | **Controls** | **Cases** | | **Controls** | **Cases** | | **Controls** | **Cases** | |
|  |  | **All** | **Self^2^** |  | **All** | **Self^2^** |  | **All** | **Self^2^** |
| **Genistein**  1st | 212 | 227 | 146 | 113 | 124 | 79 | 99 | 103 | 67 |
| 2nd | 211 | 187 | 116 | 113 | 91 | 51 | 98 | 96 | 65 |
| 3rd | 211 | 183 | 115 | 113 | 83 | 48 | 98 | 100 | 67 |
| 4th | 211 | 193 | 115 | 113 | 119 | 58 | 98 | 74 | 57 |
| **Daidzein**  1st | 212 | 247 | 166 | 113 | 115 | 79 | 99 | 132 | 87 |
| 2nd | 211 | 197 | 113 | 113 | 110 | 52 | 98 | 87 | 61 |
| 3rd | 211 | 164 | 102 | 113 | 78 | 46 | 98 | 86 | 56 |
| 4th | 211 | 182 | 111 | 113 | 114 | 59 | 98 | 68 | 52 |
| **Biochanin A**  1st | 212 | 225 | 146 | 113 | 130 | 78 | 99 | 95 | 68 |
| 2nd | 211 | 194 | 108 | 113 | 91 | 47 | 98 | 103 | 61 |
| 3rd | 211 | 181 | 120 | 113 | 104 | 62 | 98 | 77 | 58 |
| 4th | 211 | 190 | 118 | 113 | 92 | 49 | 98 | 98 | 69 |
| **Formononetin**  1st | 212 | 232 | 143 | 113 | 124 | 71 | 99 | 108 | 72 |
| 2nd | 211 | 170 | 102 | 113 | 89 | 51 | 98 | 81 | 51 |
| 3rd | 211 | 164 | 105 | 113 | 82 | 46 | 98 | 82 | 59 |
| 4th | 211 | 224 | 142 | 113 | 122 | 68 | 98 | 102 | 74 |
| **Matairesinol**  1st | 212 | 235 | 147 | 113 | 134 | 81 | 99 | 101 | 66 |
| 2nd | 211 | 180 | 115 | 113 | 85 | 49 | 98 | 95 | 66 |
| 3rd | 211 | 197 | 129 | 113 | 117 | 69 | 98 | 80 | 60 |
| 4th | 211 | 178 | 101 | 113 | 81 | 37 | 98 | 97 | 64 |
| **Secoisolarici-resinol**  1st | 212 | 220 | 145 | 113 | 125 | 80 | 99 | 95 | 65 |
| 2nd | 211 | 231 | 142 | 113 | 98 | 53 | 98 | 133 | 89 |
| 3rd | 211 | 191 | 112 | 113 | 108 | 57 | 98 | 83 | 55 |
| 4th | 211 | 148 | 93 | 113 | 86 | 46 | 98 | 62 | 47 |
| **Coumestrol**  1st | 212 | 251 | 156 | 113 | 127 | 76 | 99 | 124 | 80 |
| 2nd | 211 | 194 | 114 | 113 | 109 | 57 | 98 | 85 | 57 |
| 3rd | 211 | 172 | 116 | 113 | 91 | 60 | 98 | 81 | 56 |
| 4th | 211 | 173 | 106 | 113 | 90 | 43 | 98 | 83 | 63 |

^1^ Quartile cutpoints were calculated from series- and gender-specific nutrient intake distributions. ^2^ Self-reported cases. ^3^ alpha- and beta-Carotene.
